# Magnetic anisotropy in isotropic and nanopatterned strongly exchange-coupled nanolayers

**DOI:** 10.1186/1556-276X-7-577

**Published:** 2012-10-22

**Authors:** José Vergara, Cristina Favieres, Vicente Madurga

**Affiliations:** 1 Laboratory of Magnetism, Department of Physics, Public University of Navarre, Campus de Arrosadía s/n, Pamplona, 31006, Spain

**Keywords:** Magnetic multilayers, Exchange coupling, Magnetic anisotropy, 75.70.-i, 75.30.Gw, 81.15.Fg

## Abstract

In this study, the fabrication of magnetic multilayers with a controlled value of the in-plane uniaxial magnetic anisotropy field in the range of 12 to 72 kA/m was achieved. This fabrication was accomplished by the deposition of bilayers consisting of an obliquely deposited (54°) 8-nm-thick anisotropic Co layer and a second isotropic Co layer that was deposited at a normal incidence over the first layer. By changing the thickness value of this second Co layer (*X*) by modifying the deposition time, the value of the anisotropy field of the sample could be controlled. For each sample, the thickness of each bilayer did not exceed the value of the exchange correlation length calculated for these Co bilayers. To increase the volume of the magnetic films without further modification of their magnetic properties, a Ta spacer layer was deposited between successive Co bilayers at 54° to prevent direct exchange coupling between consecutive Co bilayers. This step was accomplished through the deposition of multilayered films consisting of several (Co_8 nm-54°_/Co_*X* nm-0°_/Ta_6 nm-54°_) trilayers.

## Background

Considerable research effort has recently been devoted to fabricating new materials that have an optimum response to electromagnetic fields in the ultrahigh frequency (UHF) range. This effort has been motivated by advancements in electromagnetic devices such as personal computers, palmtop terminals and cell phones. For instance, the recording heads of computer hard drives have demanded the use of materials that have optimum performance in the UHF range. The increasing value of the density of information stored in these drives [1] has required an increase in the rate at which information is written and read from these devices [2]. This speed can reach 2 Gbit/s, which forces the hard disk drive heads to perform optimally at frequencies on the order of several gigahertz.

In addition, the operating frequencies of the mobile phone standards (UMTS) are also in the range of frequencies of approximately 0.9 (GSM) or 2.1 GHz (3G). Furthermore, wireless Bluetooth networks operate at frequencies of 2.4 GHz, and local area wireless networks, Wi-Fi, work at 5.4 GHz. Therefore, the devices used either in cell phones or phone stations or in wireless communications systems must be adapted for use in the range of frequencies from 1 to 6 GHz.

In this scenario, the requirements for magnetic materials, usually in the form of films, that operate in the gigahertz range of frequencies are as follows [3]:

• A high value for the saturation magnetisation, *M*_S_

• A high value for the electrical resistivity, *ρ*

• The presence of an in-plane uniaxial magnetic anisotropy whose value should be controllable to fulfil the frequency requirements of the devices in which these materials are present

Different techniques have been used to induce and control the in-plane uniaxial magnetic anisotropy value in films. For example, a magnetic field has been applied during the deposition process or during a post-deposition heat treatment [4-7]. Furthermore, thin films have been deposited onto pre-patterned substrates [8-10]. Finally, off-axis deposition has also been used [11-19].

Using the previously described techniques, several works have reported control over the in-plane uniaxial magnetic anisotropy value. For instance, Li et al. [20] fabricated FeCoHf films by co-sputtering both a Fe-Co target and a Hf target. Upon increasing the Hf content, the value of the anisotropy field increased. Furthermore, Chai et al. [21] deposited CoNb/Ta multilayers and observed that the value of the anisotropy field could increase to 40 kA/m when the thickness of the Ta layer was decreased. Similarly, McMichael et al. deposited thin Co films on obliquely deposited Ta underlayers, and anisotropy fields greater than 120 kA/m were measured in the samples [22]. Furthermore, Zuo et al. [23] fabricated FeCoSi films that were partially oxidised after the samples were exposed to an oxygen flow. The values of the saturation magnetisation and the anisotropy field of these samples were larger in the samples that had a thicker ferromagnetic layer. Finally, it was recently reported that the value of the magnetic anisotropy in FeCoHfO films could be modified by changing the sputtering power [24].

Nanocrystalline Co films have previously been fabricated using the pulsed laser deposition (PLD) technique, and it has already been reported that an in-plane uniaxial magnetic anisotropy could be induced in these films by oblique deposition [25]. Particularly, for 90-nm-thick Co films, the value of the magnetic anisotropy field was relatively low (up to 8 kA/m) for samples deposited at angles of incidence up to 50°, but the value of the anisotropy field increased to 40 kA/m when the angle of incidence was increased to 55° due to the formation of nanostrings on the surface of the sample, although a further increase in the deposition angle to 60° did not modify the value of the magnetic anisotropy field. Therefore, the former abrupt change in the value of the anisotropy field in such a narrow range of deposition angles presented a drawback for the desired accurate control over the value of the anisotropy field.

In our present work, we detail a procedure to control the value of the magnetic anisotropy field, in the range of 12 to 72 kA/m, in ferromagnetic films produced using the PLD technique. This procedure was based on the deposition of bilayers and consists of the following:

• a nanopatterned Co layer deposited at an angle of 54° and

• an isotropic Co layer deposited at normal incidence (deposition angle 0°).

## Methods

Co/Co/Ta multilayers were deposited using PLD in a stainless-steel chamber (Neocera, Beltsville, MD, USA) at a base pressure of 10^−5^ mbar. We used a pulsed Nd-YAG laser (Brilliant, Quantel, Newbury, UK) with a wavelength, *λ*, of 1,064 nm; frequency of 20 Hz; and pulse duration of 5 ns. The average energy per unit area on the surface of the target was 0.2 GW/cm^2^ during the laser pulses. These targets were Co disks and Ta sheets (99.95% purity) from Goodfellow Cambridge, Ltd. (Huntingdon, UK). During the deposition process, the targets were rotated at 32 rpm to avoid excessive heating of the region irradiated by the laser. The incidence angle of the laser beam on the target surface was 45°.

**Figure 1:**
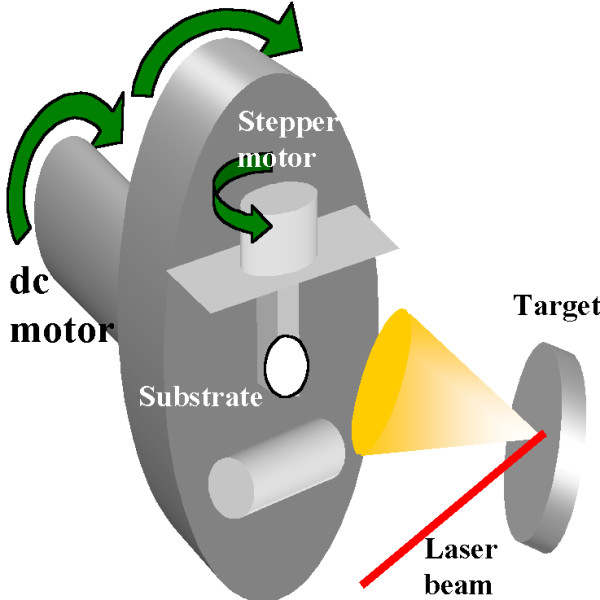
Schematic of the experimental device used for the deposition process.

The substrates were glass circles with a diameter of 7 mm and a thickness of 0.15 mm. We chose this circular geometry to avoid any in-plane magnetic anisotropy due to the shape of the substrate. The circular substrates were fixed with double-sided tape to a rectangular prism that was attached to the axis of a stepper motor configured to move in steps of 9°. Therefore, the deposition angle could be controlled from 0° (normal deposition) to 90°. This stepper motor was located on a platform that was attached to the axis of a dc motor, as shown in Figure 1. The platform, and subsequently the substrate, was rotated during the deposition process to increase the homogeneity of the deposited film. The distance between the target and the axis of the rotating substrate was 45 mm.

Using this geometry, we deposited Co/Co/Ta multilayers on a 6-nm-thick Ta buffer layer. The thickness of the initial Co layer, which was deposited at 54°, was 8 nm. The thickness of the subsequent Co layer deposited at normal incidence (0°) was changed for the different samples from 1 to 20 nm by increasing the deposition time. The 6-nm-thick Ta layer was also obliquely deposited at 54°. The thickness of each layer was monitored using a quartz microbalance.

The magnetic moment of the samples was measured using a vibrating sample magnetometer (VSM, EG&G, Princeton, NJ, USA), and the saturation magnetisation of the deposits (*μ*_0_*M*_S_ = 1.4 T) was determined through Hall effect measurements [26]. From the ratio of the magnetic moment to the saturation magnetisation, we calculated the sample volume and, consequently, the sample thickness. We used this procedure to determine the thicknesses of both the normally deposited Co layers and the obliquely deposited Co layers because the deposition rates in each case were different. The values of these thicknesses were used as input parameters in our deposition controller, which was based on a quartz microbalance, to monitor the thickness and deposition rate of the Co films. On this basis, the thickness and deposition rates of Ta could also be monitored by adjusting the values of the density and the *Z*-factor for Ta in the deposition controller.

## Results and discussion

**Figure 2:**
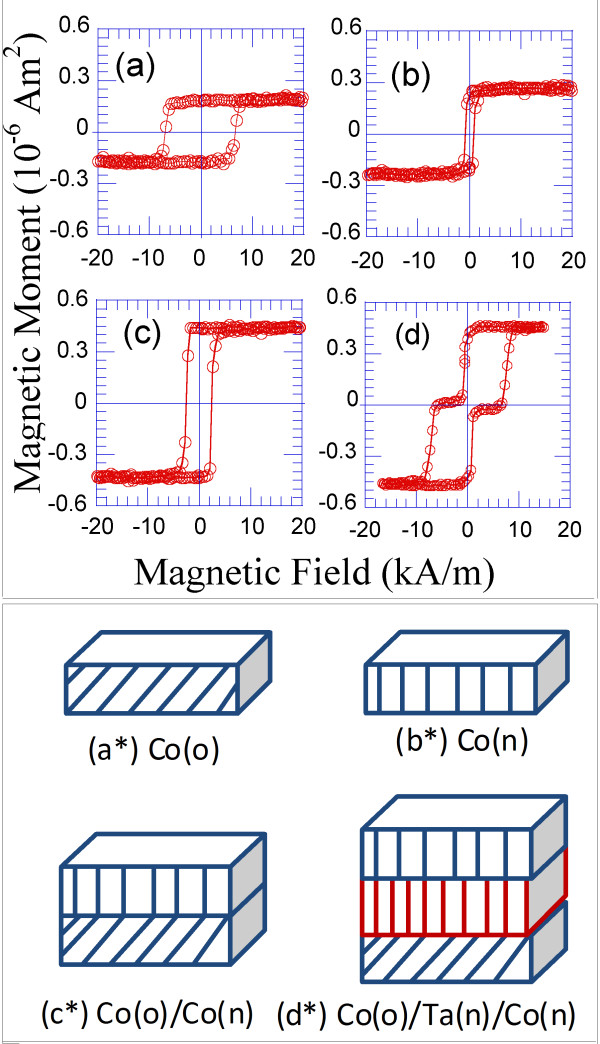
**Room-temperature hysteresis loops measured along the easy magnetisation direction for the following: (a) 4-nm-thick Co film deposited at 54°; (b) 4-nm-thick Co film that was normally deposited; (c) a bilayer consisting of two strongly exchange-coupled 4-nm-thick Co layers, where one layer was obliquely deposited at 54° and the other layer was normally deposited; and (d) a trilayer consisting of a 4-nm-thick Co layer deposited at 54°, a 6-nm-thick Ta spacer layer and a 4-nm-thick normally deposited Co layer.** In this case, the ferromagnetic Co layers are magnetically decoupled due to the effect of the Ta spacer layer. The schematic illustrations corresponding to the mentioned samples are labelled as follows: (a*) 4-nm-thick Co film deposited at 54°; (b*) 4-nm-thick Co film that was normally deposited; (c*) a bilayer consisting of a 4-nm-thick Co film deposited at 54° and a normally deposited 4-nm-thick Co film; and (d*) a trilayer consisting of a 4-nm-thick Co film deposited at 54°, a 6-nm-thick Ta spacer layer and a normally deposited 4-nm-thick Co film.

Bilayers consisting of an obliquely deposited Co layer and a normally deposited Co layer on top of the first layer were fabricated using the PLD technique, and their magnetic hysteresis loops are plotted in Figure 2.

**Figure 3:**
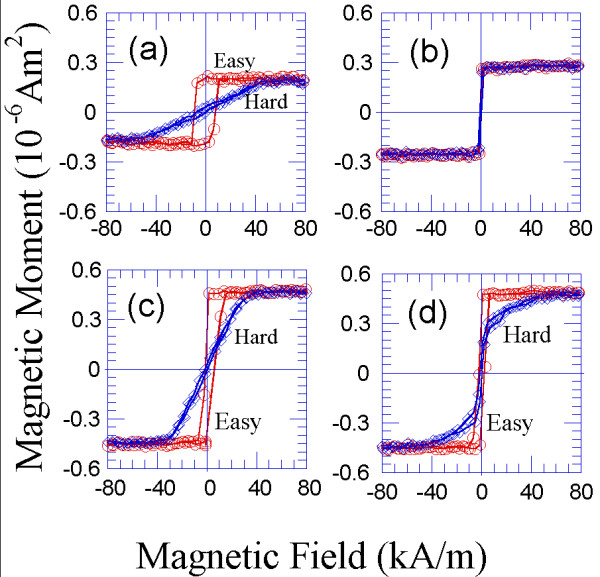
**Room-temperature hysteresis loops measured along the easy and hard magnetisation directions for the following: (a) an anisotropic 4-nm-thick Co film deposited at 54°; (b) an isotropic 4-nm-thick Co film that was normally deposited; (c) a bilayer consisting of two strongly exchange-coupled 4-nm-thick Co layers, where one layer was obliquely deposited at 54° and the other layer was normally deposited; and (d) a trilayer consisting of a 4-nm-thick Co layer deposited at 54°, a 6-nm-thick Ta spacer layer and a 4-nm-thick normally deposited Co layer.** Again, the ferromagnetic Co layers are magnetically decoupled due to the effect of the Ta spacer layer. The illustrations shown in Figure 2 also correspond to these deposits.

In this bilayer, each of the Co layers was strongly exchange-coupled to the neighbouring layer, and as a consequence of this coupling, the magnetic properties of the bilayer were the weighted average of the magnetic properties of each of the constituent layers [27]. In Figures 2a and 3a, the hysteresis loops of a 4-nm-thick Co sample deposited at 54° are shown. A coercive field along the easy magnetisation direction on the order of 7 kA/m and an in-plane uniaxial magnetic anisotropy field on the order of 56 kA/m were measured in this sample (Figures 2a and 3a, respectively). In contrast, a coercive field on the order of 1 kA/m and an isotropic behaviour were measured for the 4-nm-thick Co sample deposited at normal incidence (Figures 2b and 3b, respectively). In the bilayer fabricated from the previous layers, the coercivity was approximately 4 kA/m, and the value of the anisotropy field was 28 kA/m (Figures 2c and 3c). These magnitudes resulted from a weighted average of the magnitudes that correspond to the previous layers.

Therefore, this result illustrated an example of two strongly exchange-coupled magnetic layers that, despite exhibiting the different individual magnetic properties of each layer, behave as a single magnetic entity. The only restriction to this model is that the thickness of the resulting bilayer should be smaller than the exchange correlation length, *l*_ex_

(1) $$l_{\text{ex}}=\sqrt{\frac{A}{K_{u}}}\text{,}$$

where *A* is the exchange stiffness constant (for calculations, we used the value of 2.8 × 10^−11^ J/m that corresponds to nanocrystalline Co [28]), *K*_*u*_ (1/2 *μ*_0_*M*_S_*H*_*K*_) is the uniaxial magnetic anisotropy energy density of a particular PLD film and *H*_*K*_ is the anisotropy magnetic field. In our samples, *l*_ex_ is on the order of several tens of nanometres (20 to 50 nm) depending on the particular value of *K*_*u*_ for a given sample. A similar scenario is observed in exchange spring magnets composed of strongly exchange-coupled soft and hard magnetic layers [29-31]. In these systems, it was also demonstrated that on increasing the thickness of the soft magnetic layer beyond *l*_ex_, the soft and the hard layers decoupled magnetically [32].

Therefore, it is possible to control the value of the anisotropy field of a PLD film by changing the thickness of one of the layers. In our particular case, the thickness of the obliquely deposited Co layer will remain fixed at 8 nm. (In a previous study, we measured the value of the magnetic anisotropy field of Co films deposited by PLD at 55°, whose thicknesses varied between 1 and 100 nm. We observed that a maximum value of the anisotropy field was measured for the 8-nm-thick Co films [25].) The thickness of the layer deposited at normal incidence will vary between 1 and 20 nm. Therefore, the resulting thickness of the bilayer, which was less than 30 nm, was significantly smaller than the thickness of films typically used for UHF applications, which are on the order of 100 nm. This length is smaller than the penetration depth of the electromagnetic wave in the UHF range, which in this type of nanocrystalline material is typically 1 μm.

Therefore, to increase the volume of the magnetic material in the samples without further perturbation of their magnetic properties, we have explored the possibility of including a non-magnetic spacer layer between the neighbouring Co bilayers. To include this layer, we proceeded in the following way:

1. To test the effect of a spacer layer, we fabricated Co layers with a non-magnetic separation layer between each layer to avoid direct exchange coupling among them. We used a 6-nm-thick Ta spacer layer because a magnetic coupling between the obliquely and normally deposited Co layers was observed for smaller thicknesses of the Ta spacer layer.

2. We measured the magnetic hysteresis loops of the resulting Co/Ta/Co trilayers, where one of the Co layers was 4-nm-thick and obliquely deposited at 54° and the other layer was also 4-nm-thick but normally deposited. These results are presented in Figures 2 and 3. Because the 6-nm-thick Ta spacer layer prevented direct exchange coupling between the magnetic layers, the resulting Co_4 nm-54°_/Ta/Co_4 nm-0°_ trilayer behaved like the superposition of each of the individual Co layers, without averaging the magnetic properties in this case. Therefore, the hysteresis loops of the Co_4 nm-54°_/Ta/Co_4 nm-0°_ trilayer along the easy magnetisation axis revealed the existence of two nucleation fields that correspond to each of the Co layers (Figure 2d). However, when the magnetic field was applied along the hard magnetisation axis (of the obliquely deposited Co layer), an initial increase in the magnetisation at a relatively low applied magnetic field was observed. Furthermore, this abrupt increase was followed by a progressive increase of the magnetisation as the value of the applied magnetic field was increased to 56 kA/m, at which the saturation of the magnetisation was reached (Figure 3d). This behaviour reinforced the fact that the Ta spacer layer magnetically uncoupled, in this case, the obliquely and the normally deposited Co layers. Some schematic drawings of the normal and obliquely deposited layers and the magnetically coupled and uncoupled multilayers are shown in Figure 2.

**Figure 4:**
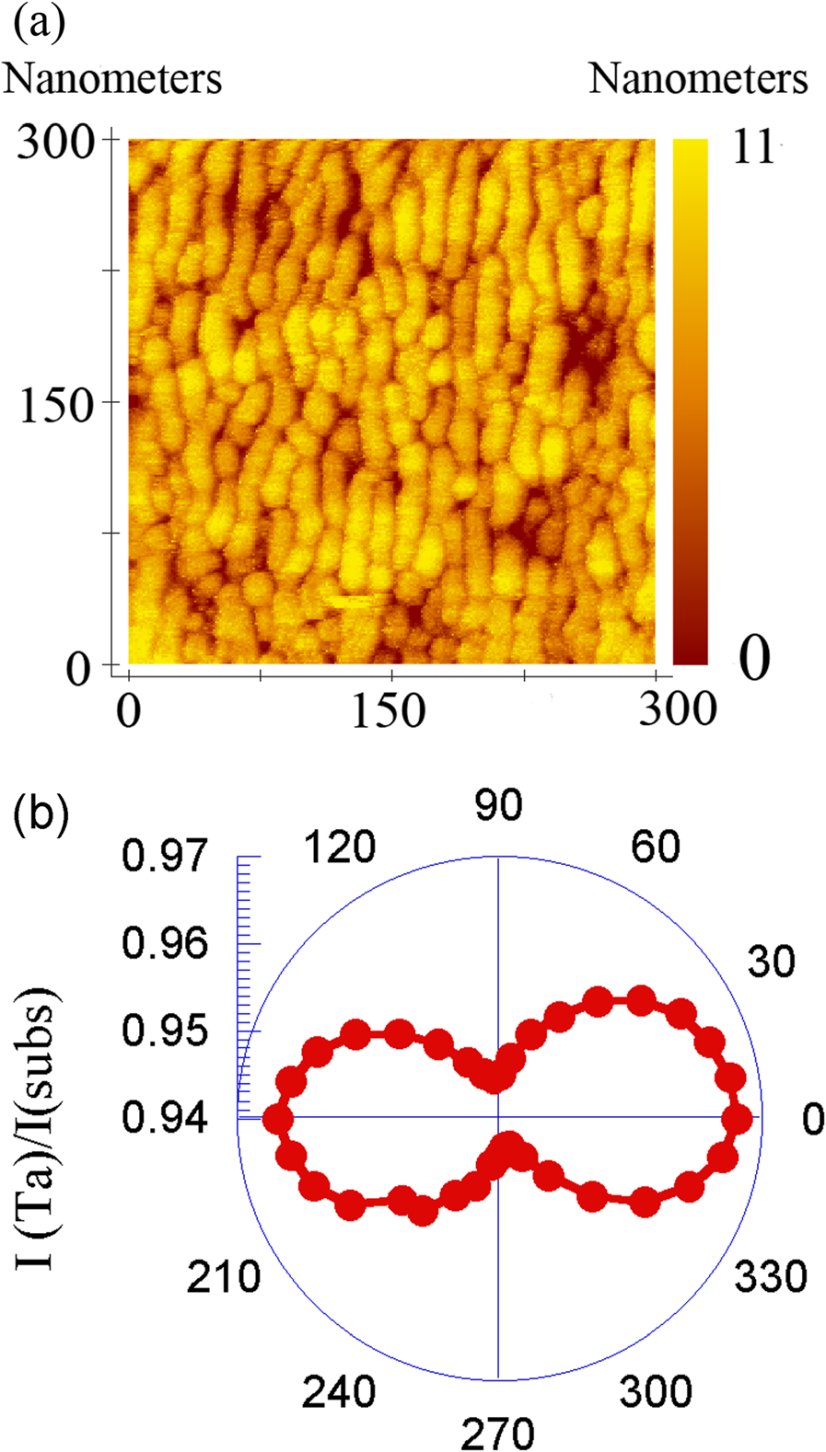
**STM micrograph of Ta layer and polar plot.** (**a**) STM micrograph of a 6-nm-thick obliquely deposited (54°) Ta layer and (**b**) a polar plot of the transmitted intensity of a polarised laser beam through different directions of the 6-nm-thick obliquely deposited (54°) Ta film, which reveals the anisotropy in the optical properties of the sample.

The use of the Ta spacer layer has therefore allowed us to deposit multilayers consisting of sets of four Co_54°_/Co_0°_/Ta_54°_ trilayers deposited onto a 6-nm-thick Ta buffer layer deposited at 54°. Thus, as indicated above, each of these trilayers consisted of an initial 8-nm-thick obliquely deposited (54°) Co layer, a second layer whose thickness varied between 1 and 20 nm that was normally deposited (0°) and a 6-nm-thick Ta spacer layer that was also deposited at 54°. Note that in this particular geometry of oblique deposition, the Ta single layers that we deposited exhibited an optical anisotropy (see Figure 4), which was likely due to the presence of nanostrings on their surfaces, which will subsequently favour the growth of Co nanostrings on them.

**Figure 5:**
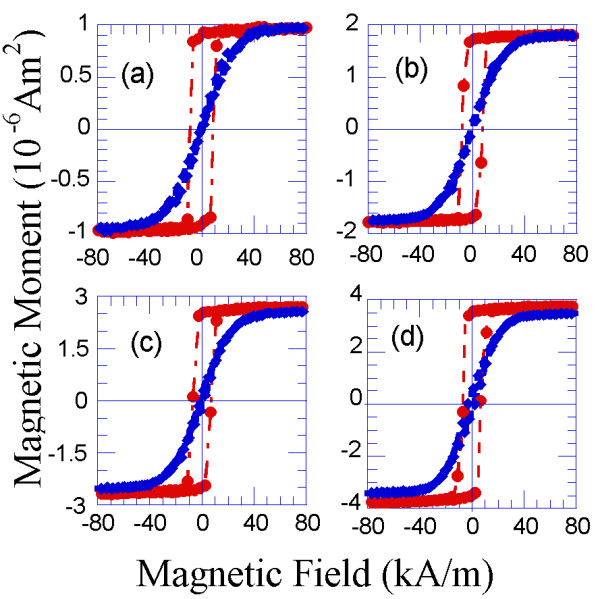
**Magnetic hysteresis loops along the easy (red) and hard (blue) magnetisation directions.** The samples consist of an increasing number of (Co_8 nm-54°_/Co_12 nm-0°_/Ta_6 nm-54°_) trilayers: one to four trilayers plotted in (**a**) to (**d**), respectively.

Therefore, the samples fabricated in this particular geometry maintained constant values of the magnetic anisotropy field and coercivity of one individual Co bilayer, whereas the volume and magnetic moment of the final sample increased by increasing the number of trilayers. This result is demonstrated in Figure 5, where the magnetic hysteresis loops of samples produced with 1 to 4 (Co_8 nm-54°_/Co_12 nm-0°_/Ta_6 nm-54°_) trilayers on a Ta buffer layer are presented.

**Figure 6:**
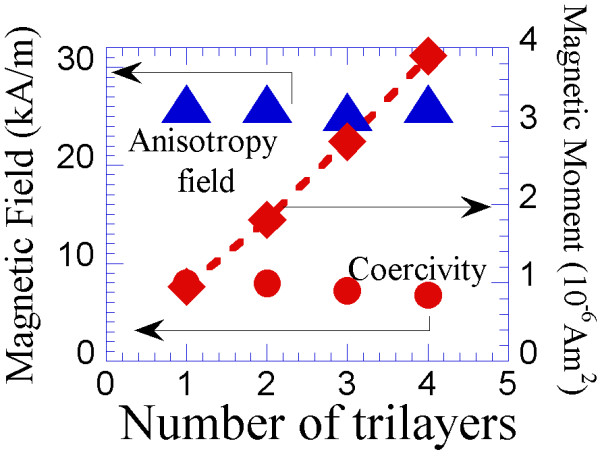
**The values of the coercive field, anisotropy field and magnetic moment of the (Co**_**8 nm-54°**_**/Co**_**12 nm-0°**_**/Ta**_**6 nm-54°**_**)**_***n***_**nanolayers.** Samples are plotted as a function of *n*, the number of trilayers, which varies from 1 to 4.

The values of the coercivity, anisotropy field and magnetic moment of each sample are specifically presented in Figure 6.

**Figure 7:**
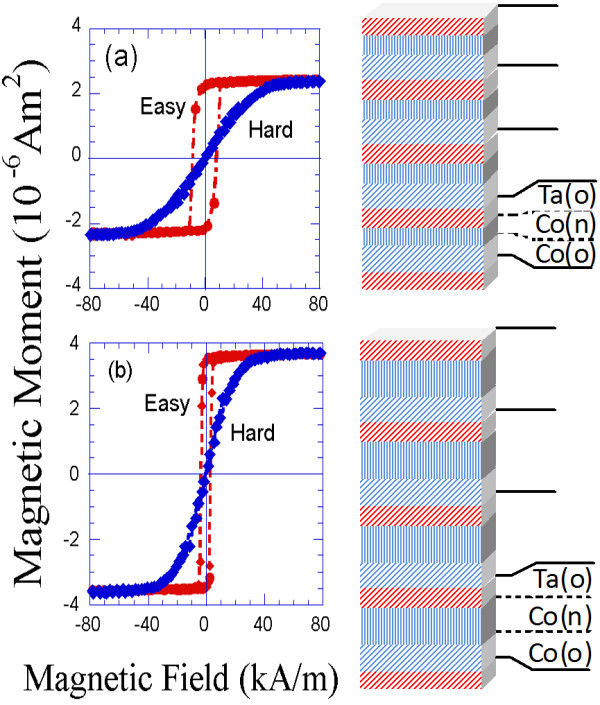
**Room-temperature magnetic hysteresis loops of two samples consisting of four trilayers and corresponding illustrations.** Each trilayer consists of an 8-nm-thick obliquely deposited (54°) Co layer; a normally deposited Co layer of variable thickness (6 nm for the sample displayed in (**a**) and 12 nm for the sample displayed in (**b**)); and an obliquely deposited (54°) 6-nm-thick Ta spacer layer. Each sample was also deposited on an obliquely deposited 6-nm-thick Ta buffer layer.

Hysteresis loops along the easy and hard magnetisation directions for different samples of the ((Co_8 nm-54°_/Co_*X* nm-0°_ /Ta_6 nm-54°_)_4_) series of nanolayers grown on an obliquely deposited (54°) 6-nm-thick Ta buffer layer are displayed in Figure 7. It is clearly shown in this figure that, on increasing the value of the thickness of the normally deposited Co layer, the value of the magnetic anisotropy of the sample decreased.

**Figure 8:**
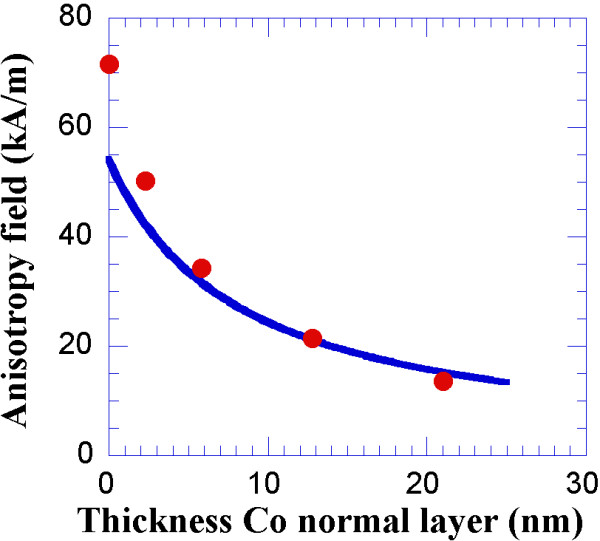
**Experimental values of the magnetic anisotropy field.** Experimental values of the magnetic anisotropy field for different trilayers as a function of the thickness of their normally deposited Co layers. The solid line is a fit to Equation 2.

Moreover, Figure 8 presents the value for the magnetic anisotropy field of our multilayers as a function of the thickness of the normally deposited Co layer.

The solid line in Figure 8 represents the fit of the magnetic anisotropy field to the prediction of the model of strongly exchange-coupled layers [27]:

(2) $$H_{K}=\frac{H_{K}\left(\text{Co}-0{}^{\circ}\right)*t_{\text{Co}-0{}^{\circ}}+H_{K}\;\left(\text{Co}-54{}^{\circ}\right)*t_{\text{Co}-54{}^{\circ}}}{t_{\text{Co}-0{}^{\circ}}+t_{\text{Co}-54{}^{\circ}}}\text{,}$$

where *H*_*K*_ is the value of the anisotropy field of either the obliquely deposited Co layer (54 kA/m for this fit) or the normally deposited Co layer (0 kA/m) and *t* is the thickness of the Co layer, deposited either at 54° (*t*_Co-54°_) or at 0° (*t*_Co-0°_).

According to this particular model, there appears to be a good agreement between the experimental and theoretical values of the anisotropy field at relatively large values of the thickness of the normally deposited Co layer. The disagreement observed at low values could be due to interface effects that are not considered within this model for strongly exchange-coupled layers. A further study of this effect will be addressed in a future work.

## Conclusions

Multilayered samples have been fabricated using a pulsed laser deposition technique. Each sample consisted of a set of four trilayers, and each trilayer consisted of different nanolayers, as indicated below:

• First, an obliquely deposited (angle 54°) 8-nm-thick Co layer, which presented an in-plane uniaxial magnetic anisotropy.

• Second, a normally deposited (angle 0°) Co layer, whose thickness varied between 1 and 20 nm, that was isotropic. These two layers were strongly exchange-coupled, and because the thickness of this resulting bilayer was smaller than the exchange correlation length for Co, the magnetic properties of the resulting bilayer were an average of the magnetic properties of the individual layers. Therefore, each bilayer behaved as a single magnetic macromolecule.

• Third, an obliquely deposited (angle 54°) 6-nm-thick Ta layer that was deposited on top of the Co bilayer described above. This Ta spacer layer prevented possible direct exchange coupling between neighbouring Co bilayers. Therefore, it was possible to increase the volume of the sample by increasing the number of trilayers without changing the magnetic properties of the Co bilayers.

In the multilayered samples, an in-plane uniaxial magnetic anisotropy was observed, and the value of the magnetic anisotropy field directly depended on the thickness of the normally deposited Co layer, which, in our case, was the only variable. Therefore, this magnetic anisotropy could be tailored by controlling the deposition time of the normally deposited Co layer. In our samples, the anisotropy field ranged from 12 to 72 kA/m.

## Competing interests

The authors declare that they have no competing interests.

## Authors’ contributions

VM, CF and JV participated from the beginning in devising the different steps of the work. CF and VM were involved in the conception of the study. VM especially participated with the experimental setup for pulsed laser deposition. JV participated with the VSM magnetic determinations and STM measurements. All the authors participated in the discussions and analysis of the results and during the preparation of the manuscript. All authors read and approved the final version of the manuscript.
